# Hydrocortisone with fludrocortisone for septic shock: a systematic review and meta‐analysis

**DOI:** 10.1002/ams2.563

**Published:** 2020-09-01

**Authors:** Ryo Yamamoto, Isao Nahara, Mitsunobu Toyosaki, Tatsuma Fukuda, Yoshiki Masuda, Seitaro Fujishima

**Affiliations:** ^1^ Department of Emergency and Critical Care Medicine Keio University School of Medicine Tokyo Japan; ^2^ Department of Anesthesiology and Critical Care Medicine Nagoya Daini Red Cross Hospital Nagoya Japan; ^3^ Department of Emergency and Critical Care Medicine Graduate School of Medicine University of the Ryukyus Okinawa Japan; ^4^ Department of Intensive Care Medicine Sapporo Medical University School of Medicine Sapporo Japan; ^5^ Center for General Medicine Education Keio University School of Medicine Tokyo Japan

**Keywords:** fludrocortisone, hydrocortisone, mortality, septic shock, shock reversal

## Abstract

**Aim:**

Combined hydrocortisone and fludrocortisone therapy for septic shock has not been evaluated with an independent systematic review. We aimed to elucidate the beneficial effects of a dual corticosteroid treatment regime involving both hydrocortisone and fludrocortisone for adult patients with septic shock on mortality.

**Methods:**

We searched the Medline, Cochrane CENTRAL, and ICHUSHI databases for reports published before April 2019. We included randomized controlled trials that compared the use of both hydrocortisone and fludrocortisone with either corticosteroid‐free or hydrocortisone‐only treatments on adult patients with septic shock. Three researchers independently reviewed the studies. The meta‐analyses were undertaken to assess primary outcome (28‐day mortality) and secondary outcomes (in‐hospital mortality, long‐term mortality, shock reversal, and adverse events).

**Results:**

Among the four studies eligible for data synthesis, we included 2,050 patients from three studies for quantitative synthesis. All studies used similar regimens (hydrocortisone and fludrocortisone for 7 days without tapering). The 28‐day mortality rate was reduced after dual corticosteroid treatment (risk ratio, 0.88; 95% confidence intervals [CI], 0.78–0.99). The heterogeneity between the studies was low (*I* ^2^ = 0%). Patients who underwent dual corticosteroid treatment had lower long‐term mortality rates (risk ratio, 0.90; 95% CI, 0.83–0.98) and higher rate of shock reversal after 28 days (odds ratio, 1.06; 95% CI, 1.01–1.12) than control patients. Adverse events (except for hyperglycemia) were similar among the treatment groups.

**Conclusions:**

The available evidence suggests that a combination of fludrocortisone and hydrocortisone is more effective than adjunctive therapy and could be recommended for septic shock.

## Graphical abstract

A systematic review and meta‐analysis were carried out on randomized controlled trials of a dual corticosteroid treatment with both hydrocortisone and fludrocortisone for adult patients with septic shock. Hydrocortisone and fludrocortisone treatment reduces the 28‐day mortality of patients with septic shock with minimum risk of adverse events. The pathophysiological mechanisms of the additional fludrocortisone and the duration of treatment should be further studied.

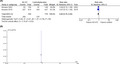

## Introduction

The beneficial effects of systematic corticosteroid treatment in adult patients with septic shock have been controversial.^1^, ^2^, ^3^, ^4^ Experimental studies have suggested the pathophysiological changes in the hypothalamic–pituitary–adrenal axis in patients with sepsis,^5^ giving rise to studies focusing on the therapeutic role of corticosteroids for sepsis and septic shock.^6^, ^7^, ^8^, ^9^, ^10^, ^11^, ^12^ Although corticosteroids have been shown to improve blood pressure,^9^ there are conflicting results on survival benefits in recent large randomized controlled trials (RCTs) and systematic reviews,^1^, ^2^, ^3^, ^4^, ^6^, ^7^, ^8^, ^10^, ^11^, ^12^ resulting in the lack of definitive recommendations in several clinical guidelines.^13^, ^14^, ^15^

Some reasons for these contradictory findings include differences in patient populations and the variation in corticosteroid treatments. Low risk‐of‐bias (RoB) RCTs recruited only patients with septic shock and investigated mortalities as their primary outcomes; however, their definition of refractory shock differed in the doses of vasopressors required.^6^, ^8^, ^11^, ^12^ The durations, amounts, and type of corticosteroids also differed; due to these inconsistencies, optimal corticosteroid treatments remain unclear.^11^, ^12^ Moreover, most systematic reviews examined particular corticosteroid therapies only through subgroup analyses.^1^, ^2^, ^4^

Among the various corticosteroid treatments, dual treatment with hydrocortisone and fludrocortisone for septic shock has shown promising results.^12^ Hydrocortisone has both glucocorticoid and mineralocorticoid activities; whereas fludrocortisone, a synthetic corticosteroid, possesses very potent mineralocorticoid activity.^16^, ^17^ Hydrocortisone has been extensively examined in sepsis, and fludrocortisone has been used for patients with aldosterone deficiency. Dual therapy using these two medications is recommended for some patients with primary adrenal insufficiency.^18^ Considering that patients with septic shock have been found to have unexpectedly low aldosterone levels due to hypothalamic–pituitary–adrenal axis abnormalities,^19^ dual treatment with hydrocortisone and fludrocortisone should be further validated as a type of corticosteroid treatment for septic shock.

Accordingly, we undertook a systematic review and meta‐analysis to identify beneficial effects of the dual treatment with hydrocortisone and fludrocortisone for patients with septic shock, when compared to treatment with placebo or hydrocortisone alone. We particularly examined mortality, vasopressor withdrawal, and adverse events (AEs).

## Methods

We report our findings in accordance with the Preferred Reporting Items for Systematic Reviews and Meta‐analyses Guidelines. The review protocol has been registered with PROSPERO (reference CRD42019139069).

### Search strategy

Three databases were searched in April 2019: Medline, Cochrane CENTRAL, and ICHUSHI. The search strategy is described in Tables S1, S2, and S3. We also evaluated the reference list of the relevant studies to identify additional sources.^1^, ^2^, ^4^

### Study selection

We included RCTs that fulfilled the following criteria: (i) full‐text publication in peer‐reviewed journals in English or Japanese, (ii) inclusion of adult patients diagnosed with septic shock, according to accepted criteria, (iii) studies comparing the use of both hydrocortisone and fludrocortisone with a corticosteroid‐free or hydrocortisone‐only comparator group.

Reviewers undertook screenings in duplicate in two stages. First, two independent reviewers (IN and MT) assessed titles and abstracts to identify potentially relevant articles. Then, the reviewers obtained full texts of articles for further review and independently assessed them. Disagreements between the two reviewers were resolved by discussion between them and a third reviewer (RY) until consensus was achieved.

### Data extraction and quality assessment

The three reviewers extracted the data independently and in duplicate using predefined data abstraction forms. The RoB was then evaluated for each outcome of individual studies using the Cochrane risk of bias assessment tool.

### Data synthesis and analysis

The primary outcome was 28‐day mortality. The secondary outcomes included in‐hospital mortality, long‐term mortality (longer than 90 days), shock reversal at day 28 defined as vasopressor withdrawal at day 28, vasopressor‐free days up to day 28, and the prevalence of AEs such as superinfection, gastrointestinal (GI) bleeding, hyperglycemia, hypernatremia, and any other events related to corticosteroid treatment. Subgroup analyses were prespecified according to duration of treatment and dose of hydrocortisone and/or fludrocortisone. Sensitivity analyses were carried out by repeating meta‐analyses, in which we defined control groups as either patients not treated with corticosteroid (placebo) or patients treated only with hydrocortisone (hydrocortisone‐only).

We used the Review Manager software (RevMan version 5.3; The Cochrane Collaboration, Copenhagen, Denmark) to undertake the meta‐analyses. We used a random‐effects model to calculate pooled effect sizes and 95% confidence intervals (CIs) for outcomes except for shock reversal at day 28, in which a fixed‐effects model was used because the effect was estimated to be in the same direction based on previous studies.^1^, ^2^, ^3^, ^4^ We presented results as risk ratios (RRs) for dichotomous outcomes and as mean differences for continuous outcomes. Heterogeneity between studies was assessed using the χ^2^‐test for homogeneity, *I* ^2^ statistic, and visual inspection of forest plots. Publication bias was also evaluated by a funnel plot. The overall certainty of evidence was rated using the Grading of Recommendations Assessment, Development, and Evaluation approach.

## Results

### Study selection

**Fig. 1:**
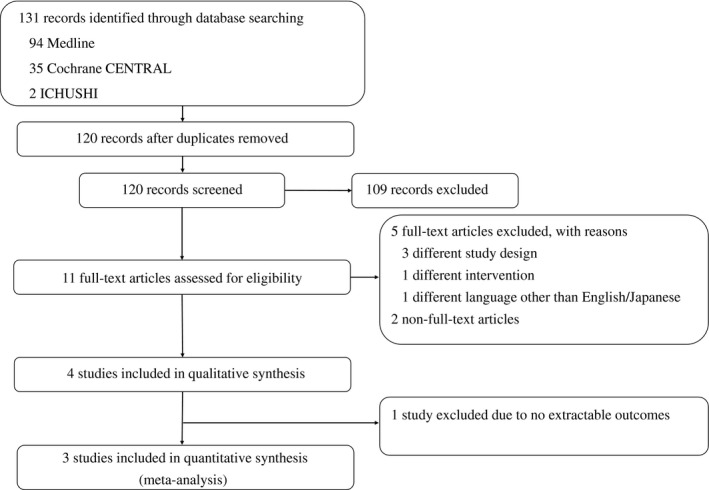
Study selection flow diagram. Among four studies eligible for data synthesis, a total of 2,050 patients from three studies were included for quantitative synthesis.

We identified 94 articles through the Medline search, 35 through the Cochrane CENTRAL search, and two articles through the ICHUSHI search. Eleven studies were considered potentially eligible; we then excluded seven after the full‐text screening. Among four studies eligible for data synthesis, we included a total of 2,050 patients from three studies for our quantitative synthesis (one study did not report any targeted outcome; Fig. 1).

### Description of eligible studies

Table S4 presents a description of the eligible studies.^6^, ^12^, ^20^, ^21^ All studies were undertaken at multiple centers, and their eligibility criteria included the requirement of vasopressors to define septic shock. All studies also used the same intervention protocol in terms of type, dose, and duration of the corticosteroid therapy; hydrocortisone was given as a 50 mg i.v. bolus every 6 h, and fludrocortisone as a 50 μg tablet once daily for 7 days (without tapering). Three of the eligible studies used placebo for the control group,^6^, ^12^, ^21^ and the other used the hydrocortisone alone therapy (a 50 mg i.v. bolus every 6 h) for the control group.^20^ One of the eligible studies reported only hematological and biochemical outcomes^21^ obtained from the same population of another included study;^6^ therefore, we did not include it in the quantitative synthesis.

### Primary outcome

**Fig. 2:**
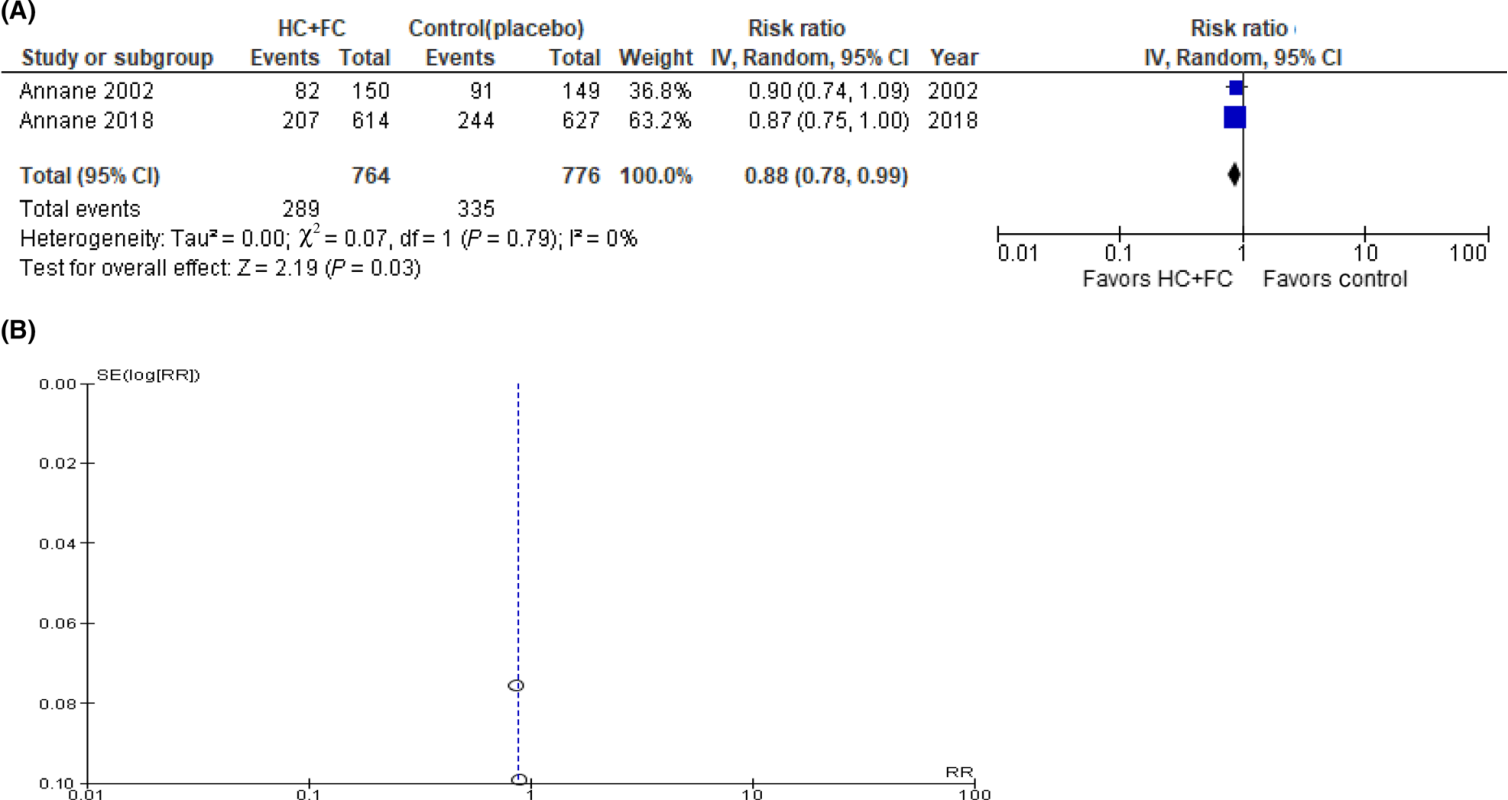
A, Forest plots of 28‐day mortality in sepsis patients who received dual corticosteroid or corticosteroid‐free treatment. B, Funnel plot of publication bias analysis. CI, confidence interval; *df*, degrees of freedom; FC, fludrocortisone; HC, hydrocortisone; IV, inverse variance; RR, risk ratio; SE, standard error.

Two studies reported 28‐day mortalities,^6^, ^12^ and our analyses showed the 28‐day mortality rate was lower in the dual corticosteroid treatment patients than in the controls, and the RR of 28‐day mortality was 0.88 (95% CI, 0.78–0.99) with low heterogeneity (*I* ^2^ = 0%, *P* = 0.79; Fig. 2). Publication bias was not estimated using the funnel plot because only two studies were included in the meta‐analysis. Prespecified subgroup analysis was not undertaken regarding the primary outcome because the duration and dose of the corticosteroid treatments were identical between the two studies. Sensitivity analysis was not applied on the primary outcome because the control groups of both studies were cortisol‐free (placebo) populations.

### Secondary outcomes

In‐hospital mortalities and long‐term mortalities were reported in three studies.^6^, ^12^, ^20^ In‐hospital and long‐term mortalities were lower in the patients treated with both hydrocortisone and fludrocortisone (RR = 0.89; 95% CI, 0.81–0.97; and RR = 0.90; 95% CI, 0.83–0.98, respectively) with low heterogeneity (Fig. S1).

**Fig. 3:**
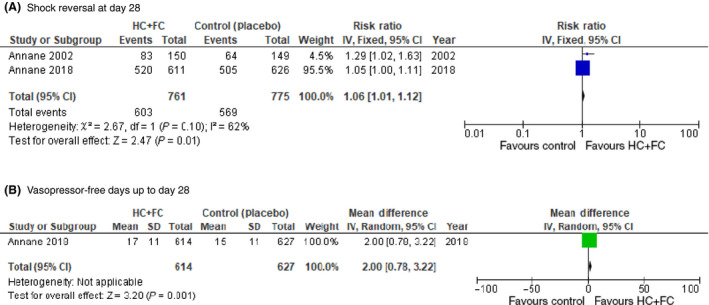
Comparison of sepsis patients who received dual corticosteroid or corticosteroid‐free treatment. A, Forest plot of shock reversal on day 28. B, Forest plot of vasopressor‐free days up to day 28. CI, confidence interval; *df*, degrees of freedom; FC, fludrocortisone; HC, hydrocortisone; IV, inverse variance; RR, risk ratio; SE, standard error.

Shock reversal at day 28 was reported in two included studies, whereas vasopressor‐free days of up to day 28 were reported in only one included study. Patients in the dual corticosteroid treatment group had a higher rate of shock reversal (RR = 1.06; 95% CI, 1.01–1.12)^6^, ^12^ and more vasopressor‐free days (mean difference = 2.0 days; 95% CI, 0.8–3.2 days)^12^ than patients in the control group (Fig. 3).

Meta‐analyses on AEs by corticosteroid treatment revealed that risks of superinfection,^6^, ^12^, ^20^ GI bleeding,^6^, ^12^ and psychiatric disorder^6^ were similar between the dual corticosteroid treatment and the control groups (Fig. S2]). The incidence of hyperglycemia was higher in patients treated with both hydrocortisone and fludrocortisone, although only one study reported hyperglycemia as an AE (Fig. S2).^12^

We did not undertake prespecified subgroup analyses on secondary outcomes because the duration and dose of corticosteroid treatments were identical among all included studies. Sensitivity analyses were carried out on in‐hospital mortality, long‐term mortality, and superinfection because the control groups comprised both a corticosteroid‐free population and a hydrocortisone‐only population. Meta‐analyses comparing the dual corticosteroid treatment with placebo showed that in‐hospital and long‐term mortalities were lower in the dual corticosteroid group than in the placebo group (RR = 0.88; 95% CI, 0.80–0.98; and RR = 0.89; 95% CI, 0.81–0.97, respectively), whereas AEs were comparable between the groups (Fig. S3).^6^, ^12^ A sensitivity analysis comparing the dual corticosteroid treatment with hydrocortisone‐only therapy revealed a higher superinfection rate in the dual corticosteroid treatment group (RR = 1.54; 95% CI, 1.05–2.26), whereas in‐hospital and long‐term mortalities were comparable between the groups (Fig. S4).^20^ Table 1 summarizes results according to control groups.

**Table 1:** Results of a systematic review of hydrocortisone and fludrocortisone for septic shock, according to control group

Outcome		Risk ratio (95% confidential interval)		
		Overall	Versus corticosteroid‐free	Versus hydrocortisone only
Mortality	28‐day mortality	**0.88 (0.78–0.99)**	**0.88 (0.78–0.99)**	N/A
	In‐hospital mortality	**0.89 (0.81–0.97)**	**0.88 (0.80–0.98)**	0.91 (0.75–1.11)
	Long‐term mortality	**0.90 (0.83–0.98)**	**0.89 (0.81–0.97)**	0.94 (0.78–1.13)
Shock reversal	Vasopressor withdrawal at day 28	**1.06 (1.01–1.12)**	**1.06 (1.01–1.12)**	N/A
	Vasopressor‐free days up to day 28 (days)	**2.0 (0.8–3.2)** ^†^	**2.0 (0.8–3.2)** ^†^	N/A
Adverse events	Superinfection	1.14 (0.85–1.51)	1.08 (0.86–1.35)	**1.54 (1.05–2.26)**
	GI bleeding	0.96 (0.66–1.39)	0.96 (0.66–1.39)	N/A
	Hyperglycemia	**1.07 (1.03–1.12)**	**1.07 (1.03–1.12)**	N/A
	Psychiatric disorders	0.33 (0.01–8.06)	0.33 (0.01–8.06)	N/A

Bold values indicate significant difference.

GI, gastrointestinal; N/A, not applicable.

^†^ Vasopressor‐free days were presented as mean difference.

### Risk of bias and summary of findings

The RoB for mortality was evaluated as “low” for all components of the Cochrane risk of bias assessment, with the exception of the “unclear risk” at “selective outcome reporting” in the study by Annane *et al*.^6^ in 2002 (Fig. S5).

The quality of evidence for each outcome is summarized in Table 2. The 28‐day and long‐term mortalities were significantly reduced by the dual therapy with both hydrocortisone and fludrocortisone with high certainty. Among the AEs associated with corticosteroid treatment, the incidence of hyperglycemia was increased by the dual corticosteroid treatment with high certainty.

**Table 2:** Summary of findings of a systematic review of hydrocortisone and fludrocortisone for septic shock

Outcomes	No. of studies	No. of patients		Effect estimates		Certainty in effect estimates^†^	Certainty assessment
		HC + FC	Control	Relative effect	Absolute effect		
				(95% CI)	(95% CI)		
28‐day mortality	2	289/764 (37.8%)	335/776 (43.2%)	RR 0.88	52 fewer per 1,000	⨁⨁⨁⨁	
				(0.78 to 0.99)	(4 fewer to 95 fewer)	High	
Long‐term mortality (90 days to 1 year)	3	478/1,009 (47.4%)	548/1,040 (52.7%)	RR 0.90	53 fewer per 1,000	⨁⨁⨁⨁	
				(0.83 to 0.98)	(11 fewer to 90 fewer)	High	
Shock reversal (at day 28)	1	603/761 (79.2%)	569/775 (73.4%)	RR 1.06	44 more per 1,000	⨁⨁⨁	Imprecision
				(1.01 to 1.12)	(7 more to 88 more)	Moderate	
Superinfection	3	266/1,009 (26.4%)	242/1,039 (23.3%)	RR 1.14	33 more per 1,000	⨁⨁	Borderline inconsistency and imprecision
				(0.85 to 1.51)	(35 fewer to 119 more)	Low	
GI bleeding	2	50/764 (6.5%)	53/775 (6.8%)	RR 0.96	3 fewer per 1,000	⨁⨁	Serious imprecision
				(0.66 to 1.39)	(23 fewer to 27 more)	Low	
Hyperglycemia	3	547/614 (89.1%)	520/626 (83.1%)	RR 1.07	58 more per 1,000	⨁⨁⨁⨁	
				(1.03 to 1.12)	(25 more to 100 more)	High	
Psychiatric disorders	3	0/150 (0.0%)	1/149 (0.7%)	RR 0.33	4 fewer per 1,000	⨁⨁	Serious imprecision
				(0.01 to 8.06)	(6 fewer to 47 more)	Low	

CI, confidential interval; FC, fludrocortisone; GI, gastrointestinal; HC, hydrocortisone; RR, risk ratio.

^†^ Certainty in effect estimates was assessed with five domains: study limitations, inconsistency, indirectness, imprecision, and publication bias.

## Discussion

We undertook the systematic review and meta‐analysis to evaluate the effects of dual corticosteroid treatment in terms of clinical outcomes in patients with septic shock. Although previous systematic reviews had shown conflicting results, we found a reduced mortality with high certainty: 28‐day, in‐hospital, and long‐term (later than 90 days) mortalities were reduced by treatment with both hydrocortisone and fludrocortisone.

The main difference between the current study and other meta‐analyses is the fact that we examined only the effects of the dual corticosteroid treatment for septic shock. The idea behind the addition of fludrocortisone to hydrocortisone, used as glucocorticoid replacement therapy in patients with adrenal insufficiency, is to enhance the mineralocorticoid activity.^16^, ^17^ Mineralocorticoids are known to affect salt and water balance, whereas glucocorticoids preferentially affect sugar metabolism and exhibit sex hormone activities,^16^ suggesting that mineralocorticoids would play a role in fluid retention among patients with septic shock. The biological activity of mineralocorticoids is mediated by the mineralocorticoid receptor (MR),^16^ which exists in various organs, such as the kidneys, cardiovascular, immune, and central nervous systems.^22^ Potential immune effects of mineralocorticoids through non‐renal MR have been suggested,^16^ and animal studies found an association between sepsis and the downregulation of the MR in endothelial cells.^23^ Mineralocorticoid supplementation lowered IL‐6 levels, hastened shock reversal, and improved survival.^24^, ^25^ Some clinical studies also revealed inappropriately low aldosterone levels in patients with septic shock, suggesting an impaired adrenal synthesis of aldosterone, which might be associated with increased mortality.^19^, ^26^

The meta‐analyses on the secondary outcomes found that the incidence of AEs was not increased by the dual corticosteroid treatment, except for hyperglycemia, which is consistent with a systematic review examining all types of corticosteroid therapies for sepsis.^1^ In that study, the risks for hyperglycemia, hypernatremia, and neuromuscular weakness were similarly increased by corticosteroid treatment, whereas the incidence of superinfections, GI bleeding, and psychiatric disorders remained similar to those in control patients. Considering that MRs are expressed in monocytes and macrophages that undergo a pro‐inflammatory polarization in response to mineralocorticoids,^27^ pathophysiological immunomodulatory changes by the additional mineralocorticoid treatment should be further examined.

Fludrocortisone use was optional in a previous version of the Surviving Sepsis Campaign guidelines,^28^ and it was removed from the most recent guidelines in 2016.^13^ Two recent systematic reviews evaluated heterogeneity in types of corticosteroid treatments, and did not find a credible effect of the specific type of corticosteroid treatment.^1^, ^4^ However, these analyses did not examine the direct association between the dual corticosteroid treatment and clinical outcomes, and based on our results additional use of fludrocortisone would be considered more than just an adjunctive therapy.

There are several limitations in this study. We found only four eligible studies and included only two in the meta‐analysis for the primary outcome,^6^, ^12^ in part because the additional fludrocortisone has not been extensively examined and because we considered only RCTs. However, our search strategy used a wide variety of search terms and the eligibility criteria were wide enough to capture an article by Laviolle *et al*.^21^ that was not included in the recent systematic reviews.^1^, ^2^, ^3^, ^4^

Another limitation of this study is the fact that the control group in the meta‐analyses consisted of both corticosteroid‐free and hydrocortisone‐only populations, which could hamper the interpretation of our results. Although some secondary outcomes differed according to the definitions of the comparator group, the reduced 28‐day mortality by the dual corticosteroid therapy resulted only from the comparison with placebo.

Moreover, all eligible studies used the same treatment regimen. Although different doses might affect the results, the doses used were consistent with those used in replacement therapy for primary adrenal insufficiency.^18^, ^29^ Given that a study on different hydrocortisone‐only treatment durations for septic shock revealed no differences in outcomes between 3‐day and 7‐day regimens,^30^ a shorter regimen of the dual corticosteroid treatment should be investigated.

Finally, one of the included studies reporting 28‐day mortality was carried out approximately 20 years ago. As the quality of care for sepsis has significantly improved and the definition of sepsis has changed in the last two decades, the efficacy of dual corticosteroid treatment should be further assessed among patients diagnosed using the current criteria.

## Conclusions

This systematic review clarified that hydrocortisone and fludrocortisone treatment reduces the 28‐day mortality of patients with septic shock with minimum risk of AEs. The pathophysiological mechanisms of the additional fludrocortisone and the duration of treatment should be further studied.

## Disclosure

Approval of the research protocol: N/A.

Informed consent: N/A.

Registration no.: PROSPERO No. CRD42019139069.

Conflict of interest: SF has received personal fees from Asahi Kasei Japan and Takeda Pharmaceutical, grants from Chugai Pharmaceuticals, Daiichi‐Sankyo, Otsuka Pharmaceutical, Pfizer, Astellas Pharma, Shionogi, and Teijin Pharma outside the submitted work. YM reports grants from JIMRO and personal fees from MSD, Japan Blood Products Organization, and Asahi Kasei Pharma outside the submitted work.

## Supporting information


**Fig S1.** Forest plots of in‐hospital and long‐term mortalities.

**Fig S2.** Forest plots of adverse events.

**Fig S3.** Sensitivity analyses comparing the dual corticosteroid therapy with placebo.

**Fig S4.** Sensitivity analyses comparing the dual corticosteroid therapy with hydrocortisone‐only regimen.

**Fig S5.** Risk of bias summary.

**Table S1.** Search strategy overview.
**Table S2.** Medline search strategy.
**Table S3.** Cochrane CENTRAL search strategy.
**Table S4.** Characteristics of the included studies.
