# Supplementary figures and images for: Hydrocortisone with fludrocortisone for septic shock: a systematic review and meta‐analysis
Source: Acute Med Surg. 2020 Sep 1;7(1):e563. doi: 10.1002/ams2.563 (PMC7507448; doi:10.1002/ams2.563)

## Slide 1
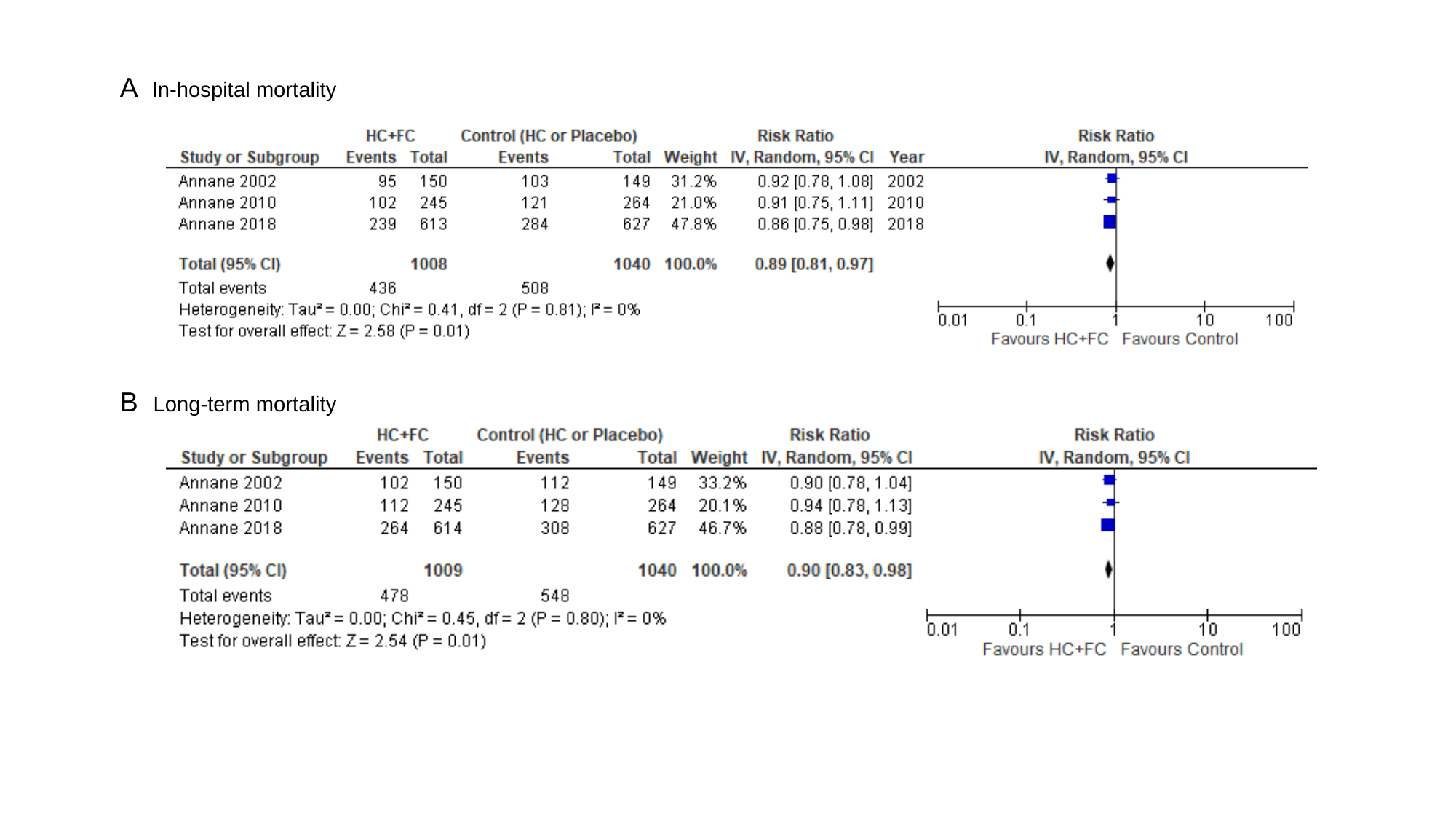

A In-hospital mortality
B Long-term mortality

Supplement: Supplementary file 1 — Fig S1. Forest plots of in‐hospital and long‐term mortalities. [file AMS2-7-e563-s001.pptx]

## Slide 1
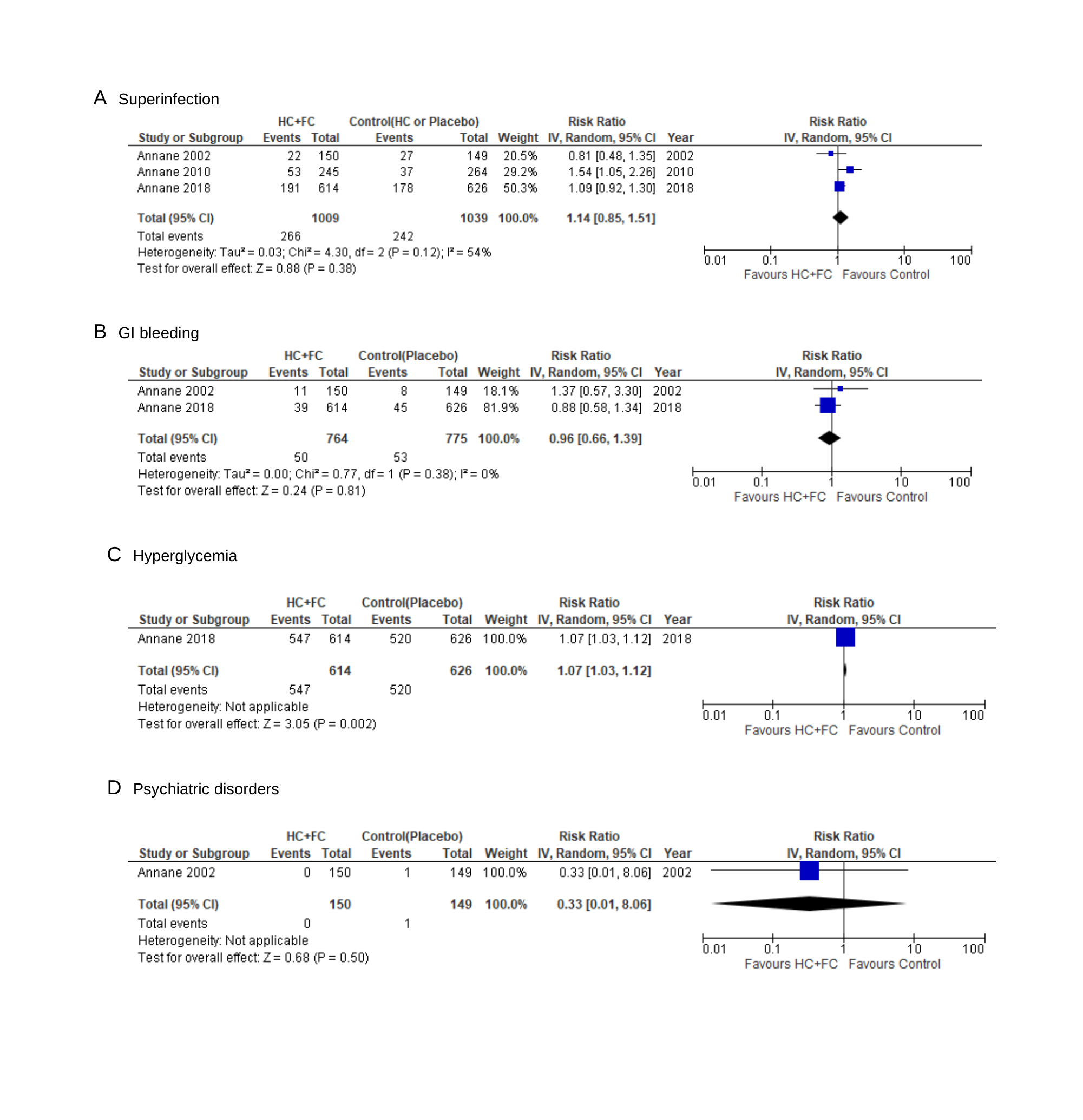

A Superinfection
B GI bleeding
C Hyperglycemia
D Psychiatric disorders

Supplement: Supplementary file 2 — Fig S2. Forest plots of adverse events. [file AMS2-7-e563-s002.pptx]

## Slide 1
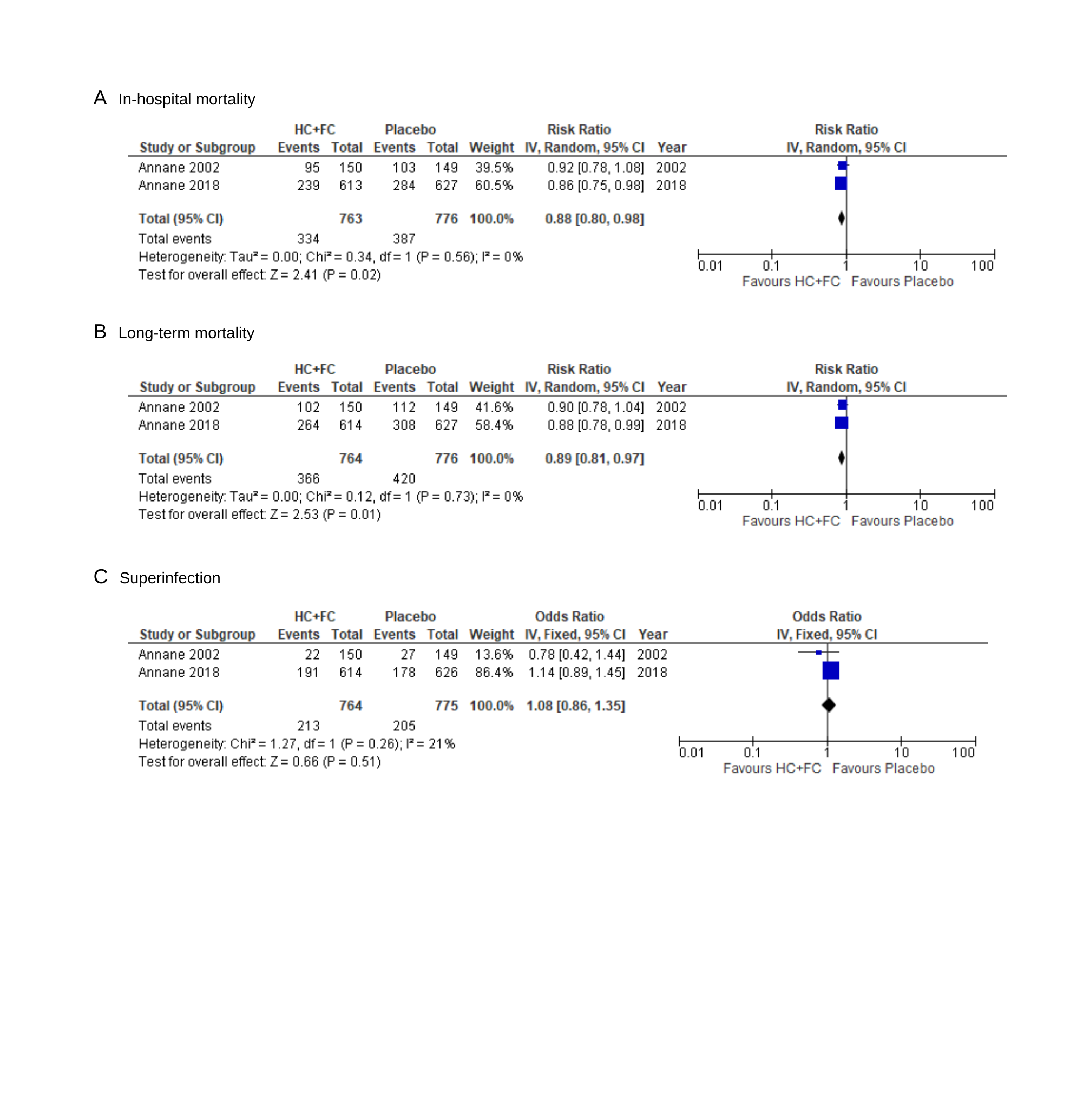

A In-hospital mortality
B Long-term mortality
C Superinfection

Supplement: Supplementary file 3 — Fig S3. Sensitivity analyses comparing the dual corticosteroid therapy with placebo. [file AMS2-7-e563-s003.pptx]

## Slide 1
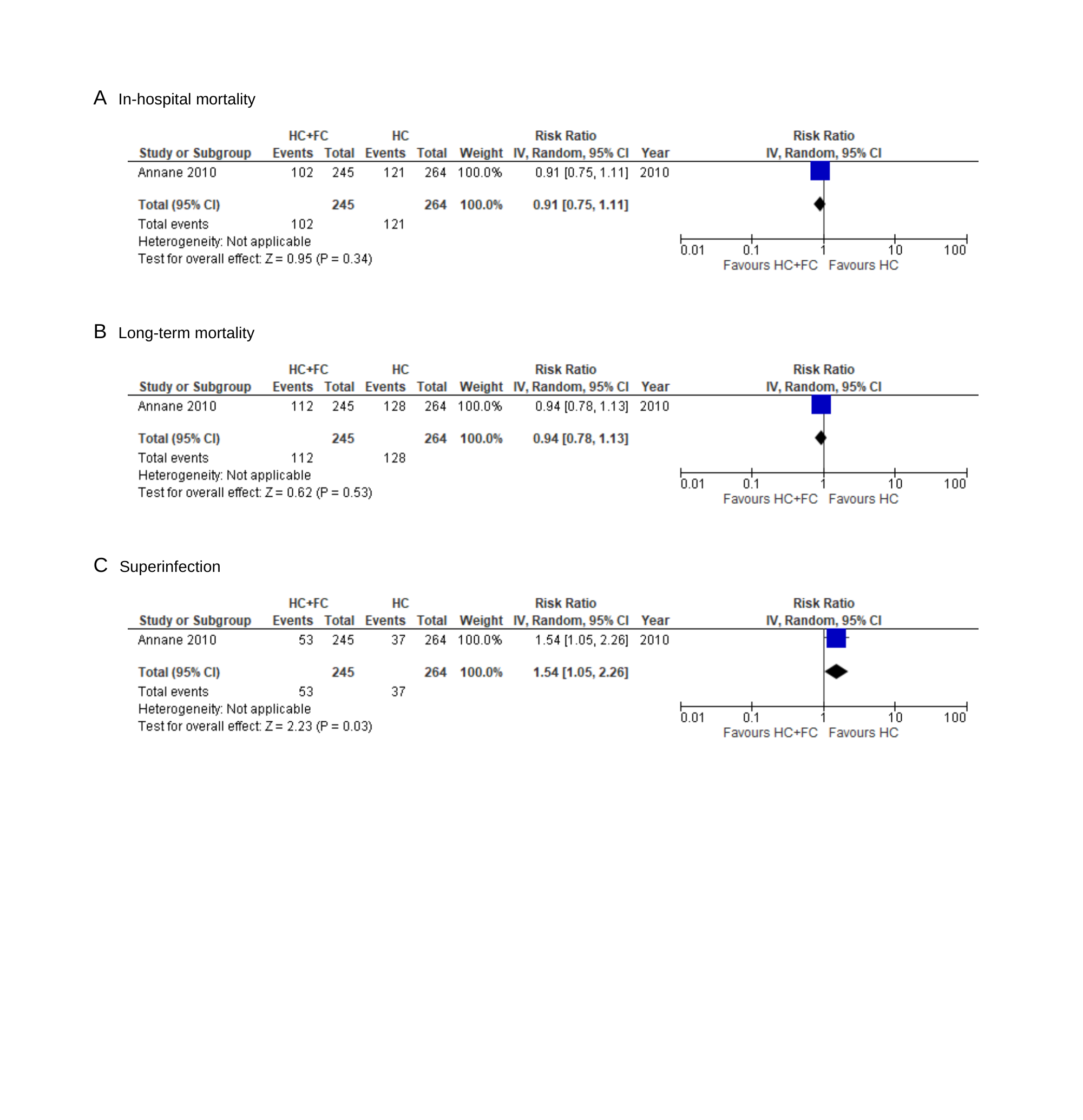

A In-hospital mortality
B Long-term mortality
C Superinfection

Supplement: Supplementary file 4 — Fig S4. Sensitivity analyses comparing the dual corticosteroid therapy with hydrocortisone‐only regimen. [file AMS2-7-e563-s004.pptx]

## Slide 1
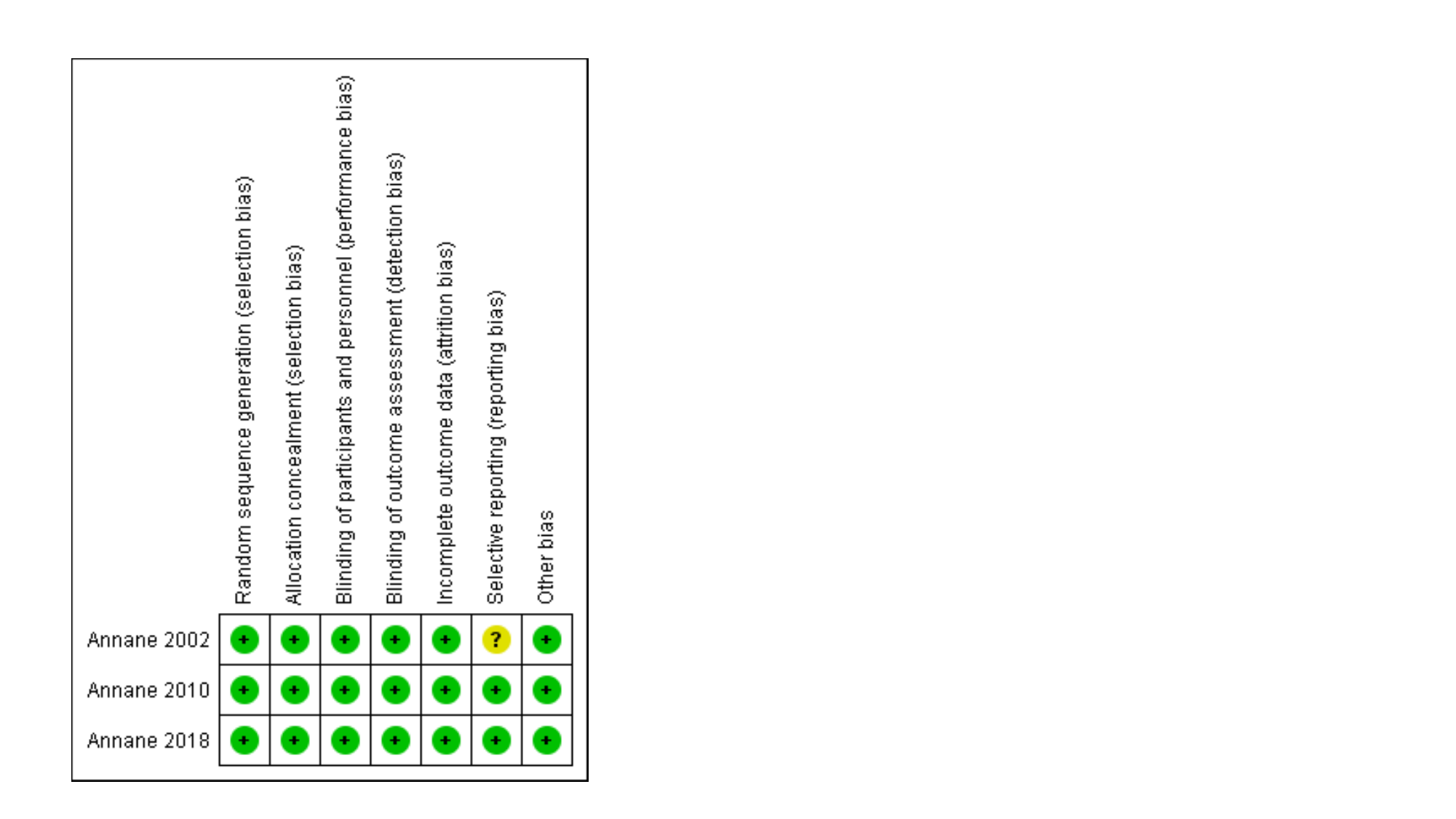

Supplement: Supplementary file 5 — Fig S5. Risk of bias summary. [file AMS2-7-e563-s005.pptx]
