# Supplementary material for: Hydrocortisone with fludrocortisone for septic shock: a systematic review and meta‐analysis
Source: Acute Med Surg. 2020 Sep 1;7(1):e563. doi: 10.1002/ams2.563 (PMC7507448; doi:10.1002/ams2.563)
Supplement: Supplementary file 6 — Table S1. Search strategy overview. Table S2. Medline search strategy. Table S3. Cochrane CENTRAL search strategy. Table S4. Characteristics of the included studies. [file AMS2-7-e563-s006.docx]

**Table E1. Search strategy overview**

| The search strategy included Medical Subject Headings search terms and keywords.  Search terms were selected for following two domains. |
| --- |
| 1) patient populations (e.g., “sepsis,” “systemic inflammatory response syndrome,” “organ failure,” or “critical illness”) |
| 2) dual corticosteroid treatment as intervention (e.g., “hydrocortisone” AND “fludrocortisone”) |

**Table E2. Medline search strategy**

| #1 | "Sepsis"[Mesh] OR sepsis[tiab] OR septic[tiab] | 192238 |
| --- | --- | --- |
| #2 | "Systemic inflammatory response syndrome"[mesh] OR "Systemic inflammatory response syndrome"[tiab] OR SIRS[tiab] | 123788 |
| #3 | "Multiple Organ Failure"[mesh] OR " Organ Failure"[tiab] OR MOF[tiab] OR "organ dysfunction"[tiab]　OR “MODS”[tiab] | 37655 |
| #4 | "Critical illness"[mesh] OR "critical care"[mesh] OR "intensive care units"[mesh] OR stressed[tiab] OR "critically ill"[tiab] OR "critical care"[tiab] OR "intensive care"[tiab] | 262456 |
| #5 | virus[tiab] OR "Virus Diseases"[Mesh] OR viral[tiab] | 1283555 |
| #6 | #1 OR #2 OR #3 OR #4 OR #5 | 1714391 |
| #7 | "Fludrocortisone"[Mesh] OR "fludrocortisone acetate" [Supplementary Concept] OR “Fludrocortisone”[tiab] OR "Mineralocorticoids"[Mesh] OR "Mineralocorticoids" [Pharmacological Action] OR "Mineralocorticoids"[tiab] | 12284 |
| #8 | "Hydrocortisone"[Mesh] OR "hydrocortisone acetate" [Supplementary Concept] OR “Hydrocortisone”[tiab] OR "Glucocorticoids"[Mesh] OR "Glucocorticoids" [Pharmacological Action] OR “Glucocorticoids”[tiab] | 261188 |
| #9 | #7 AND #8 | 4505 |
| #10 | (randomized controlled trial[pt] OR controlled clinical trial[pt] OR randomized[tiab] OR placebo[tiab] OR drug therapy[sh] OR randomly[tiab] OR trial[tiab] OR groups[tiab] NOT (animals [mh] NOT humans [mh])) | 3888206 |
| #10 | #6 AND #9 AND #10 | 94 |

Searched on April 28^th^, 2019

**Table E3. CENTRAL search strategy**

| #1 | MeSH descriptor: [Sepsis] explode all trees | 3998 |
| --- | --- | --- |
| #2 | "sepsis":ti,ab,kw | 10248 |
| #3 | septic:ti,ab,kw | 4108 |
| #4 | MeSH descriptor: [Virus Diseases] explode all trees | 25707 |
| #5 | virus:ti,ab,kw | 29011 |
| #6 | viral:ti,ab,kw | 18805 |
| #7 | MeSH descriptor: [Systemic Inflammatory Response Syndrome] explode all trees | 4328 |
| #8 | "systemic inflammatory response syndrome":ti,ab,kw | 1020 |
| #9 | SIRS:ti,ab,kw | 676 |
| #10 | MeSH descriptor: [Multiple Organ Failure] explode all trees | 384 |
| #11 | "organ failure":ti,ab,kw | 2807 |
| #12 | "organ failures":ti,ab,kw | 174 |
| #13 | MOF:ti,ab,kw | 141 |
| #14 | "organ dysfunction":ti,ab,kw | 1416 |
| #15 | "organ dysfunctions":ti,ab,kw | 114 |
| #16 | MODS:ti,ab,kw | 247 |
| #17 | MeSH descriptor: [Critical Illness] explode all trees | 1845 |
| #18 | MeSH descriptor: [Critical Care] explode all trees | 1918 |
| #19 | MeSH descriptor: [Intensive Care Units] explode all trees | 3260 |
| #20 | stressed:ti,ab,kw | 958 |
| #21 | "critically ill":ti,ab,kw | 5961 |
| #22 | "critical care":ti,ab,kw | 3515 |
| #23 | "intensive care":ti,ab,kw | 20327 |
| #24 | #1 or #2 or #3 or #4 or #5 or #6 or #7 or #8 or #9 or #10 or #11 or #12 or #13 or #14 or #15 or #16 or #17 or #18 or #19 or #20 or #21 or #22 or #23 | 85864 |
| #25 | MeSH descriptor: [Hydrocortisone] explode all trees | 5701 |
| #26 | hydrocortisone:ti,ab,kw | 8875 |
| #27 | "hydrocortisone acetate":ti,ab,kw | 310 |
| #28 | MeSH descriptor: [Glucocorticoids] explode all trees | 4256 |
| #29 | glucocorticoid:ti,ab,kw | 3400 |
| #30 | glucocorticoids:ti,ab,kw | 5943 |
| #31 | #25 or #26 or #27 or #28 or #29 or #30 | 15865 |
| #32 | MeSH descriptor: [Fludrocortisone] explode all trees | 104 |
| #33 | fludrocortisone:ti,ab,kw | 205 |
| #34 | "fludrocortisone acetate":ti,ab,kw | 19 |
| #35 | MeSH descriptor: [Mineralocorticoids] explode all trees | 45 |
| #36 | mineralocorticoid:ti,ab,kw | 1018 |
| #37 | mineralocorticoids:ti,ab,kw | 73 |
| #38 | #32 or #33 or #34 or #35 or #36 or #37 | 1181 |
| #39 | #31 and #38 | 283 |
| #40 | #24 and #39 | 33 |

Searched on April 30^th^, 2019. Pubmed and CENTRAL search strategies were translated to Japanese and used as ICHUSHI search strategy.

**Table E4. Characteristics of the included studies**

| **Author** | **Publication year** | **Total patients** | **Study setting** | **Patient eligibility** | **Corticosteroid treatment as intervention** | **Duration of intervention** | **Control** | **Study outcomes** |
| --- | --- | --- | --- | --- | --- | --- | --- | --- |
| Annane et al.^6^ | 2002 | 300 | 19 ICUs in France | 18 year or older, septic shock (sBP < 90 mmHg for at least 1 h despite fluid replacement and vasopressor), arterial lactate > 2 mmol/L, mechanical ventilation | Hydrocortisone hemisuccinate powder 100 mg, IV bolus every 6 h  Fludrocortisone tablet 50 μg, nasogastric tube every 24 h | 7 days | Placebo | 28-day mortality, 1-year mortality, in-hospital mortality, vasopressor withdrawal at day 28, superinfection, GI bleeding, psychiatric disorders |
| Annane et al.^20^ | 2010 | 509 | 11 ICUs in France | Adults, severe sepsis, SOFA score of 8 or more, vasopressor to maintain sBP >90 mmHg or MBP >60 mmHg, | Hydrocortisone hemisuccinate powder 100 mg, IV bolus every 6 h  Fludrocortisone tablet 50 μg, nasogastric tube every 24 h | 7 days | Hydrocortisone hemisuccinate powder (100 mg), IV bolus every 6 h | 180-day mortality, in-hospital mortality, vasopressor-free days up to day7, superinfection |
| Laviolle et al.^21,a^ | 2012 | 299 | 19 ICUs in France | 18 year or older, septic shock (sBP < 90 mmHg for at least 1 hour despite fluid replacement and vasopressor), arterial lactate > 2 mmol/L, mechanical ventilation | Hydrocortisone hemisuccinate powder 100 mg, IV bolus every 6 h  Fludrocortisone tablet 50 μg, nasogastric tube every 24 h | 7 days | Placebo | Hematological and biochemical variables (e.g., hemoglobin, leukocyte count, urinary sodium, urinary potassium) |
| Annane et al.^12^ | 2018 | 1241 | 65 ICUs in France | Adults, septic shock; SOFA score ≥ 3 for ≥2 organs for ≥6 h, vasopressor to maintain sBP ≥ 90 mmHg or MBP ≥ 65 mmHg for ≥6 h | Hydrocortisone hemisuccinate powder 100 mg, IV bolus every 6 h  Fludrocortisone tablet 50 μg, nasogastric tube every 24 h | 7 days | Placebo | 28-day mortality, in-hospital mortality, 90-day mortality, vasopressor withdrawal at day 28, vasopressor-free days up to day 28, superinfection, GI bleeding, hyperglycemia |

sBP, systolic blood pressure; GI bleeding, gastrointestinal bleeding; SOFA score, sequential organ failure assessment score; MBP, mean blood pressure. ^a^Laviolle et al in 2012^21^ examined the same population of the study by Annane et al in 2002^6^ and reported only hematological and biochemical outcomes. This study was not included in meta-analyses.
